# An individually randomized controlled trial to determine the effectiveness of the Women for Women International Programme in reducing intimate partner violence and strengthening livelihoods amongst women in Afghanistan: trial design, methods and baseline findings

**DOI:** 10.1186/s12889-018-5029-1

**Published:** 2018-01-22

**Authors:** Andrew Gibbs, Julienne Corboz, Mohammed Shafiq, Frozan Marofi, Anna Mecagni, Carron Mann, Fazal Karim, Esnat Chirwa, Charlotte Maxwell-Jones, Rachel Jewkes

**Affiliations:** 1 Gender and Health Research Unit, South African Medical Research Council, Pretoria, South Africa; 2 Women for Women International, Kabul, Afghanistan; 3 Women for Women International, London, UK; 4 Eureka, Kabul, Afghanistan

**Keywords:** Afghanistan, Trial, Economic empowerment, Gender transformation, Intervention, Baseline

## Abstract

**Background:**

Intimate Partner Violence (IPV) is the most common form of violence in conflict and post-conflict settings, but there are few evaluations of interventions to prevent IPV in such settings.

**Methods:**

The Women for Women International (WfWI) intervention is a year-long combined economic and social empowerment intervention for marginalized women survivors of conflict. Primarily, it seeks to support women to achieve four key outcomes: women earn and save money; women improve their health and well-being; women influence decisions in their homes and communities; women connect to networks for support. The organization recognizes Violence Against Women and Girls (VAWG) as a significant barrier to women’s empowerment and expects to see reduction in VAWG, and specifically IPV, as part of building women’s social and economic empowerment. This program is being quantitatively evaluated through an individually randomized control trial amongst women in Afghanistan, with a 24-month follow up. A comparison of baseline characteristics of participants is also included as well as a discussion of implementation of the baseline research.

**Discussion:**

There is a high demand amongst Afghan women for such interventions, and this posed challenges in completing the randomization and baseline. In addition, the complex security situation in Afghanistan also posed challenges. However, despite these issues, recruitment was successfully achieved and the arms were balanced on socio-demographic measures. The evaluation will contribute to the limited evidence base on interventions to prevent IPV in conflict-affected settings.

**Trial registration:**

NCT03236948. Registered 28 July 2017, retrospectively registered.

## Background

In conflict and post-conflict settings, intimate partner violence (IPV) is the most common form of violence that women experience, despite non-partner sexual violence and other forms of violence being more often focused on [1, 2]. There is also increasing evidence that high rates of IPV are likely to continue after conflict has ended [3–7] and are fueled by the enduring impact of conflict [8]. IPV and other violence experienced by women and girls need to be prevented in conflict-affected settings, as much as this must be done in more peaceable contexts.

Until fairly recently, reducing IPV among conflict-exposed populations was not very high on the peace-building or development agenda. As a result, few interventions to reduce IPV have been tested in these settings [1]. The interventions tested have generally combined economic empowerment of women with or without some form of other elements, including gender-focused ones. In conflict-affected communities in Uganda, a women’s economic empowerment intervention showed no impact on IPV experience, but the addition of a short male partner involvement intervention showed some positive (but non-significant) improvements in relationships [9]. Similarly, in Afghanistan, an economic-only intervention showed increases in women’s economic participation and mobility, but showed no impact on women’s status within the home [10]. In post-conflict Ivory Coast, a women’s savings group intervention had gender dialogue sessions added to it, which showed positive trends in outcomes [11]. The positive effects of interventions combining gender transformative/social empowerment components with economic interventions is reflected in the wider IPV prevention research in non-humanitarian settings [12, 13]. More research is needed to develop interventions that are appropriate and effective in challenging contexts.

Afghanistan has experienced more than four decades of conflict and has a deeply patriarchal gender regime based on conservative cultural practices and conservative Islamic interpretations of men’s and women’s roles. Women have very little power, violence against women and girls is endemic, and harmful traditional practices are widespread. These include honour killings, early and forced marriage of girls [14], bride price (*walwar* or *sherbaha*^1^), *baad* (the giving of girls to a disputant party to settle a debt or conflict) and *badal* (women and girls exchanged between families for marriage). Women’s options for leaving abusive relationships are highly constrained: women’s freedom of movement and access to social safety nets are highly limited; and women who ‘run away’ are regarded as having attempted to commit the ‘moral crime’ of *zina* (which implies either adultery or having sexual relations with somebody out of wedlock) regardless of whether there is any evidence or not. Women survivors of sexual violence have also been accused of committing *zina.* If caught, women would be punished either through the formal system (jail) or through informal systems and there have been many reports of women being abused at the hands of mobs. Women have also been subjected in abusive investigatory techniques by the police, including (scientifically invalid) ‘virginity testing.’^2^

The use and acceptability of violence against women and girls in Afghanistan is widespread. The 2015 Afghanistan Demographic and Health Survey (AFDHS) found that 80% of women and 72% of men (for both groups, married and aged 15 to 49) agreed that it was justified for a woman to be beaten by her husband in at least one of five circumstances, particularly if a woman goes out of the house without telling her husband [15]. An early estimate on women’s experiences of IPV in Afghanistan from research conducted with 4700 women in 16 (of 34) provinces found that 73.9% of women reported psychological IPV, 52.4% of women reported experiencing physical IPV ever, and 39.3% of women experienced physical IPV in the previous year [16]. The study found risk factors for experiencing IPV included being in a forced marriage, being in a polygamous marriage, being under age 15 years old and married, having gender inequitable attitudes, and living in a rural area [16].

More recent data from the 2015 AFDHS indicates that 56% of ever married women aged 15 to 49 reported having ever experienced any type of spousal violence (emotional, physical or sexual) and 52% reported having experienced at least one of these forms of violence in the past 12 months [15]. Spousal physical IPV is the most common type of violence, with 51% of women reporting ever having had experienced physical violence (e.g. being slapped, pushed or shaken, punched, kicked, dragged, choked, burned or attacked with a weapon, or having their arm twisted or hair pulled). Of those women having reported spousal physical violence in the past 12 months (45.8%), 26% sustained subsequent physical injuries [15]. Fewer women reported ever having experienced any kind of spousal emotional violence (37%), or sexual violence (7%), though it is likely sexual violence is under-reported. The Afghanistan DHS found a wide range of risk factors for spousal violence, including women being employed but not being paid in cash (i.e. receiving goods or other non-cash earnings), lower education of both women and their spouses, husbands’ controlling behaviour, a family history of domestic violence, and living in a rural area [15]. There was also wide variation by Province in the prevalence of lifetime IPV, ranging from 6% in Helmand province and 7% in Badakhshan Province, to 92% in Ghor and Herat Provinces [14].

This paper describes the methods for an evaluation of an economic strengthening and social empowerment intervention, developed and used for 15 years by the international non-governmental organization Women for Women International (WfWI) in Afghanistan. We report the methods in terms of the SPIRIT (Standard Protocol Items: Recommendations for Interventional Trials) 2013 Checklist [17], and compare baseline characteristics of participants. The trial is an individually randomized control trial with endline follow up 24 months after baseline.

## Methods/design

The trial is conducted as part of the “What Works To Prevent Violence? A Global Programme on Violence Against Women and Girls (VAWG)”, funded by the UK Government’s Department for International Development (DFID).^3^ The Global Programme is funding research on 16 interventions in 13 countries to assess the effectiveness of the interventions in prevention of VAWG. The work is conducted in Africa, the Middle East and Asia.

This trial is being led by the South African Medical Research Council working through local research agencies in Afghanistan, and the intervention is being delivered as per normal practice by WfWI.

### Objectives

The aim of this study is to evaluate the effectiveness of the Women for Women International (WfWI) intervention in reducing IPV experienced by married Afghan women and improving their mental health.

1. The primary objective is to determine whether exposure to the WfWI intervention is effective in reducing experience of intimate partner violence and improving the mental health of women in the programme.
2. The secondary objective it to determine whether exposure to the WfWI intervention is effective in improving women’s income and savings, and creating more equitable gender attitudes, including reducing the acceptability of IPV.

### Trial design

The study is a two-arm, individually randomized controlled trial. Intervention arm participants receive the WfWI intervention (i.e. WfWI’s core programme), a combined social empowerment and livelihoods strengthening intervention, alongside a US$120 cash transfer over 12 months (US$10 per month). This cash transfer is a standard part of WfWI’s core programme to cover training-related expenses, contribute to household needs and help women to start to build savings. Control arm participants are given $10 per interview to motivate assistance in the study and as reimbursement.

### Sample size

A pilot study was conducted in August 2016 with 100 women who were already part of the WfWI intervention to assess the questionnaire, as well as provide an estimate of physical IPV experience in the past 12 months. We found many of the women already enrolled in the WfWI intervention were not currently married, in fact only 37/100 were. Among these, past year physical IPV prevalence was 32.4% and past year severe IPV prevalence was 27%. At the time, this corresponded to published data, which estimated 39.3% of women had experienced past year physical IPV [16].

We used an assumption of seeing an effect size of 0.3 difference between the 12 month prevalence of physical IPV at endline in the control and intervention arms. This is the effect size seen in several recent evaluations of effective violence prevention interventions [18, 19]. We assume that the 12 month control arm IPV prevalence will be 32% and severe IPV 27%. Using Stata, with power set at .80 and alpha at 0.05, sample size per arm was estimated to be 337 for past year physical IPV, and 422 for past year severe physical IPV. Based on our calculations for the percentage of women recruited into the intervention being currently married, and the potential loss to follow-up, the target number of participants was 1477.

### Study setting

The provinces and districts selected for the research were those in which WfWI planned to work in 2016 and 2017 during the recruitment phase. These were established in enrollment plans for the intervention designed at the start of each year, and the research was fitted in around these plans. As per previous years of implementation in Afghanistan, communities in which WfWI implemented their programming were selected through community level assessments, and coordination with district government authorities, to ensure the most appropriate communities would be included. Security concerns also factored into selection of communities, with the aim of ensuring security for WfWI staff members as well as the intervention participants. Due to the introduction of the RCT in 2016, additional criteria for community selection included ensuring a sufficient number of women were eligible to enroll in both the intervention and control arms of the study.

The evaluation was conducted in two provinces, Kabul and Nangarhar. In Kabul, three districts were selected and in Nangarhar, two districts. WfWI was working in additional provinces at the time but we chose to work in two provinces for the study design for pragmatic reasons. In each district, a central village was identified with a training centre from which WfWI could run the intervention. Depending on the size and structure of the village, women also came from surrounding areas.

### Recruitment

Once communities are selected by WfWI, WfWI field staff undertook community mobilization activities to explain the objectives of the programme and the research to community and religious leaders and obtain their support for implementation. Information was then shared in villages through community meetings and Friday Mosque prayers. Women interested in participating were asked to assemble on a scheduled date and location.

Women were individually recruited into the study. WfWI staff members explained WfWI’s core programme, while research staff members explained the research study and randomization process. Participants were screened for eligibility before being enrolled into the study.

### Eligibility criteria

The eligibility criteria for the study mostly followed the entry criteria for WfWI. The regular selection criteria for the WfWI intervention were that participants should be at the lowest economic level possible, earning less than a US$1.25 a day, they should be unemployed, not in school and not involved in a similar programme. Women incapacitated due to mental illness or very severe disability would not be eligible for the intervention, as they otherwise would not be able to fully benefit by engaging in the training sessions (e.g. if blind they would be unable to observe the training materials, or if deaf they would be unable to hear the content of the training) or by doing income generating work. Women should be willing to participate in the programme and attend all training classes and not have previously participated in the programme. Preference was given to women who are mothers or heading households, irrespective of marital status. Women were encouraged to secure support from family members for their involvement to better facilitate their participation, although this was not always possible.

Two additional criteria due to the research were added: 1) restricting the age of women to 18 and 45 years due to issues of consent for those under 18 year olds; and 2) increasing the proportion of currently married women in the sample than is general WfWI practice because in Afghanistan, it is only feasible to ask IPV questions of currently married/previously married women. The age restriction also reduced the likelihood of mother-in-laws being recruited into the study alongside their daughter-in-laws. (A pilot study found that having two members of the same household in the study led to concerns about maintaining privacy about the study).

### Intervention

**Fig. 1:**
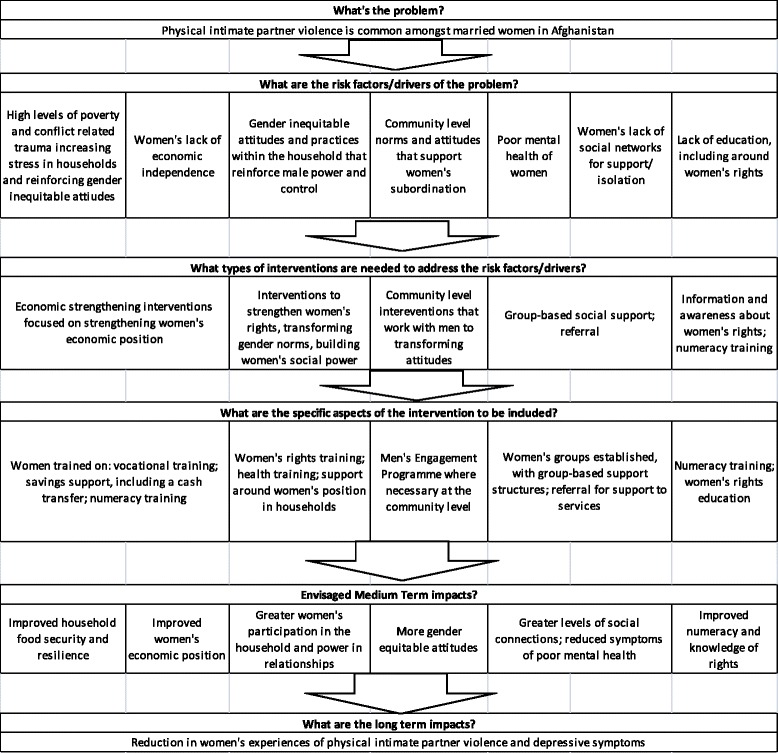
Theory of Change: Women for Women International Programme, specifically for IPV reduction

The intervention has been developed and refined since 1993 by WfWI and is an approach to intervening in complex settings in alignment with the needs of women who have been denied access to education, have been negatively affected by conflict and who seek inclusion and recovery., also referred to as WfWI’s core programme, has been implemented by WfWI in eight countries***.*** The theory of change for the intervention is shown in Fig. 1 and is focused specifically on reducing IPV.

In all the villages, activities are delivered by locally hired staff from the community who speak local dialects. Staff use a range of methods to deliver the curriculum including storytelling, role-playing, or discussion questions. Due to the typically very low levels of literacy of participants in Afghanistan, trainers and staff tend to use verbal methods. Sessions are approximately 90 min long, delivered over a year, to adapt to women’s availability and constraints on their time (such as care work).

Component 1: Informational Training: critical information about savings, health, rights, and networking is delivered through key modules as part of the year-long programme:

- The value of women’s work: This module focuses on supporting women to overcome stereotypes and inequities that prevent them from gaining economic self-sufficiency. This module educates women about division of labour, household financial management, and the different types of opportunities for income generation that are available to them.
- Health, wellness and violence against women. This module focuses on building women’s understanding of: health as an asset; health as a human right; health as an essential criterion to undertake other productive activities; women’s bodies and how to care for them; prevention, treatment, and management of key health issues; safe space to discuss sensitive topics, including conflict, sexual and gender based violence; and referrals to health services.
- Rights, family and community decision-making. This module covers human and legal rights, and what women as individuals- and as a group - can do to exercise their own rights in their families and communities. It addresses women and family law including inheritance, control of assets, marriage, divorce and child custody, and domestic violence and rape.
- Social networks and safety-nets. This module covers the value of women working together in groups and/or in social networks; building effective networks; planning for group advocacy; managing leadership issues; negotiation and conflict management (within the household).

Component 2: Skill Building: the sessions that fall under skill building are delivered through hands-on training and practice over the year-long programme and focus on building basic skills in:

- Numeracy. This is provided for women who need it so that women can learn to count, add/subtract, and use money. All women participants in Afghanistan receive this component.
- Basic business skills. Women learn and practice basic business skills needed for self-employment, group businesses or employment. It covers bookkeeping, selling, planning and marketing their products, and is followed by three sessions on cooperatives looking at the benefits and different types of cooperatives including their structures.
- Vocational skills. WfWI conducts market assessments to identify the best income generation activities for women, given their resources, education and other constraints. Specific training in Afghanistan is focused on animal husbandry, tailoring, gardening and knitting.

Component 3: Resource provision: To complement the informational training and skill building, women receive financial support, referrals and opportunities for saving through the 12 months:

- Women receive a monthly conditional cash transfer of US$10 per month (total received US$120). There is no stipulation about how women should spend this month. Some women choose to save this money to help them establish their own businesses. Others use it to cover basic needs such as food, clothing and medicine.
- WfWI provides women participants with a range of referral services. A list of referral services is developed. Where women need additional support, they are referred through to services.
- In Afghanistan, WfWI has introduced an adapted version of the Self-Help Group, an informal grouping of women who set up a small ‘bank’ in which they save together. This is not a compulsory savings programme, but many groups decide to start them as part of the programme.

Component 4: Connections to other women, local women’s networks and global supporters: Women go through the programme in classes of 25 and begin the programme with the same group with whom they graduate. This provides a safe space for socially excluded women to learn new skills, share experiences and receive support.

Women for Women International also runs a range of complementary programmes to promote sustainable change and a supportive, enabling environment for women.

Men’s engagement activities: Recognising how women’s participation and ability to affect change in their lives is often shaped by men in the household and at the community level, WfWI has developed a men’s engagement programme (MEP). The goal of the programme is to work with men and male opinion leaders to change gender attitudes and generate an openness to new ideas, such that it is possible to implement the WfWI intervention with women. In Afghanistan, this is a 24 session intervention with male religious and community leaders over three months. The focus of this training is on family rights in Islam. Selected graduates of this programme are then trained to lead “stepdown” discussion groups with men in their local communities to discuss issues around women’s rights, multiplying the reach of the initial training. The men discuss topics that affect their communities and their daily lives. The curriculum is underpinned by Islam, quoting verses from the Qur’an to underscore specific aspects of women’s rights.

The MEP is being implemented in one of the six villages for the trial as per the practice of WfWI to selectively implement the MEP in selected communities on a case-by-case basis. Through the community assessment process, WfWI find some communities show resistance to WfWI’s core programme. WfWI prioritizes those communities for the MEP intervention, as a strategy to reduce resistance and enable support for the programme.

### Outcomes

While traditional evaluations of interventions define one primary outcome, complex interventions, such as this, have increasingly defined multiple primary outcomes that are equally important [19–21]. The primary and secondary outcomes are detailed in Table 1 with questionnaire items used to evaluate outcomes, an example per outcome, and hypothesized direction of change. The trial has three primary outcomes:

- Past year experience of physical IPV amongst currently married women
- Past year experience of severe physical IPV amongst currently married women
- Women’s past week depressive symptoms

**Table 1:** Primary and secondary outcomes for the trial

	Number of items and origins	Example question and response categories	Scaling	Alpha	Hypothesized direction
Primary outcomes					
Past year experience of physical IPV amongst currently married women	Five items ask about married women’s experience of physical IPV. The scale is based on the WHO’s multi-country study of IPV [25].	(Q) In the past 12 months how many times has your husband hit you with a fist or with something else which could hurt you? (A) Never, once, few, many	A positive response to one or more items coded as yes		Decrease
Past year experience of severe physical IPV amongst currently married women	Same scale as above. Severe physical IPV is defined as experiencing more than one item of the five, or experiencing any one item more than once, creating a dichotomous measure equivalent to more than once experiencing physical IPV.	(Q) In the past 12 months how many times has your husband slapped you or thrown something at you which could hurt you? (A) Never, once, few, many	Two or more items responded to as ‘once’ or responding to any single item as “few”, or “many”.		Decrease
Women’s past week depressive symptoms	Depressive symptoms are assessed using the Center for Epidemiological Studies Depression scale (CES-D), comprising 20 items asking about depressive symptoms in the past week [26].	(Q) During the past week I had crying spells (A) Rarely or none of the time; some or little of the time; moderate amount of time; most or all of the time	Mean score created; higher mean’s more depressive symptoms	0.90	Decrease
Secondary outcomes					
Household Food Insecurity in past 4 weeks	Three items comprising the Household Hunger Scale, developed for global use and comparability [27].	(Q) In the past 4 weeks, how often was there no food to eat of any kind in your house because of a lack of money? (A) Never, Rarely, Sometimes, Often	Mean score created; higher indicates more food insecurity	0.94	Decrease
Financial shock resilience	One item asks about ability to mobilise money in an emergency. This measure was developed for use in South Africa, and has been used in Asia.	(Q) If you had an emergency at home and needed 500 Afghani, how easy would you say it would be to find the money? (A) Very easy, fairly easy, somewhat difficult, very difficult	Recoded into binary of very or fairly easy and somewhat difficult or very difficult		Decrease
Women’s monthly income	A single item asks about earnings in the past month.	(Q) Considering all the money you earned from jobs or selling things, how much did you earn last month?	Mean		Increase
Women’s total savings	A single item asks women the total value that they have in savings.	(Q) How much money have you got in savings?	Mean		Increase
Life satisfaction	Life satisfaction is assessed using four-items derived from the Satisfaction With Life Scale [28]. This has been used across South Asia [29].	(Q) In most ways my life is close to my ideal (A) Strongly agree, agree, disagree, strongly disagree	Mean; higher means less satisfied	0.90	Decrease
Past four week suicidal ideation	A single item assesses thoughts of suicide in the past month, with a binary response possible.	(Q) In the past four weeks, has the thought of ending your life been in your mind? (A) Yes, No	Binary yes/no		Decrease
Women’s gender attitudes	This scale was developed locally from discussions with Afghan’s before being tested in Pakistan. A series of 11 questions ask about gender attitudes that individual women hold.	(Q) I think it is a good thing for a young wife in my family to be beaten to teach her how to behave properly (A) Strongly disagree; disagree; agree; strongly agree	Mean score; higher score indicates less gender equitable attitudes	0.87	Decrease
Married women’s participation in household decision making	Five items are asked about women’s ability to participate in household decisions, based on the WHO Multi-Country Study on Domestic Violence [25].	(Q) In the last three months, how often your views been listened to on matters concerning the children and their schooling or work in your home? (A) Never; sometimes; often	Mean score; higher scores indicate more participation	0.77	Increase
Past year emotional IPV amongst currently married women	Seven items ask about experiences of emotional abuse by the husband.	(Q) In the past 12 months how many times has your husband insulted you or made you feel bad about yourself? (A) Never; once; few; many	Mean score; higher score indicates more emotional violence		Decrease
Perceptions of husband cruelty amongst currently married women	Five items ask about married women’s perceptions of her husband and his attitudes and relationship towards her.	(Q) My husband is very strict and controlling. (A) Strongly disagree; disagree; agree; strongly agree	Mean score: higher scores indicate more cruelty	0.88	Decrease
Mother in law and sibling abuse:	Two items: A single item assesses whether mother-in-laws have hit the woman in the past 12 months and another item assesses whether siblings have hit the woman in the past 12 months, an affirmative response to either item would indicate abuse.	(Q) In the last 12 months were you slapped, hit or beaten by your mother-in-law?. (A) Never, sometimes, often	A positive response to one or both items coded as yes		Decrease
Perceptions of mother-in-law cruelty.	For married women who currently live with their mothers-in-law, six items ask about their relationship and the mother-in-law’s attitudes towards her.	(Q) My mother-in-law can frighten me (A) Strongly disagree; disagree; agree; strongly agree	Mean score: higher scores indicate more cruelty	0.84	Decrease

The secondary outcomes for the study reflect the multiple objectives of the intervention and fall into four categories: economic outcomes; mental health and wellbeing; gender attitudes, and power and violence outcomes.

Secondary economic outcomes are:

- Past four week household food insecurity;
- Financial shock resilience;
- Women’s past month earnings; and
- Women’s total savings.

Secondary mental health and wellbeing outcomes are:

- Current life satisfaction; and
- Past four week suicidal ideation.

Secondary gender attitudes, and power and violence outcomes are:

- Women’s gender attitudes;
- Past year emotional IPV amongst currently married women;
- Perceptions of husband cruelty amongst currently married women;
- Past year mother-in-law and sibling abuse; and
- Perceptions of mother-in-law cruelty amongst currently married women.

### Data collection

Quantitative data were collected through face-to-face structured interviews using paper and pencil by trained female fieldworkers. Questionnaires were translated from English into Dari and Pashto through a process of translation and back translation, and piloted by the fieldwork team. Data collectors conducted individual interviews with women in private locations.

**Fig. 2:**
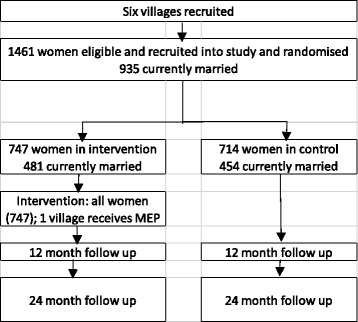
Evaluation flowchart

The participant timeline is set out in Fig. 2. Baseline data collection was completed between October 2016 and February 2017. Follow-ups are 12 months and 24 months after baseline.

### Assignment of interventions

Women were individually randomized into the intervention and control arms. Assignment of women into the intervention and control group was done through women drawing coloured balls from an opaque bag or box after enrollment into the study.

While interventions such as this are often randomized at the cluster (village) level into intervention and control arms to avoid contamination [22], pilot work showed that women may mix in public places but did not tend to talk about the types of things covered by the WfWI programme. Given the intensive education content and focus on skills building, we determined that significant contamination was unlikely. Given the levels of insecurity in Afghanistan, this methodology reduced the number of villages where access and research would have to be negotiated, reducing security risks. Randomisation occurred on the day after recruitment (two villages) or at the time of recruitment (all other villages).

In one village, WfWI ran its men’s engagement programme, as detailed above. Villages are not randomized to receive this: it is provided in villages where it is perceived work would be difficult without buy-in from the men in the community.

### Data management and analysis

Questionnaires were double-entered into SPSS, discrepancies between the entered data were identified, and answers were verified through checking the paper questionnaires and corrected. The same process will be undertaken for both follow ups.

The data will be cleaned by the study statistician. One dataset will be compiled for analysis of key variables based on the three data collections: baseline, follow up at the end of the programme (12 months from baseline), and follow up 12 months after programme graduation (24 months from baseline). The data will be described by presenting frequencies for categorical variables and means for scale and sub-scales, with 95% confidence intervals by study arm.

The main analysis for outcomes of intimate partner violence will be conducted on a sub-set of the sample who are currently married at endline. For depression, it will be conducted on the entire sample. Intention-to-treat (ITT) analyses will be conducted. A *per protocol* analysis will also be conducted as a secondary analysis (i.e. including just those women who attended more than 75% of WfWI’s core programme). Cross tabulation and chi-square tests will be used to determine whether there are differences in demographics between the treatment and control group. The primary analysis will be carried out by fitting Generalized Linear Mixed Models (GLMMs) with a term for treatment arm [23]. For outcomes using scales, the difference in the scores between baseline and 12 months, and baseline and 24 months will be assessed. For each of the scales, a linear mixed model will be fitted with fixed terms for treatment arm. The scores will be directly calculated from the component items, while the scales will combine the items using weights determined by carrying out a principal component analysis on the items at baseline, and using the loadings for the first principal component as the weight for each item. These analyses will firstly be carried out on the ITT population and then repeated for the per protocol population.

### Ethics

The research is structured according to the WHO’s guidance on doing research around violence against women [24]. The trial has been designed to ensure that the project does not expose potential of harm to participants and to respond to any emotional or psychological harm that may result from the questions that will be asked in this study.

If participants become distressed during the research or the intervention, the team has in place a series of referral mechanisms to appropriate support. Given the challenges of working Afghanistan and limited availability of services, specific referrals vary from village to village but include government health services and NGOs specialising in working with women survivors of violence.

Participants provided thumbprints as informed consent to participate in the study given the incredibly low levels of literacy. Personal data will be stored separately from data, and no identifying information is collected on the questionnaires. Unique identifying codes link questionnaires with participant information for tracking purposes at 12 and 24 months.

Ethical approval for the study was given by the South African Medical Research Council (EC034–11-2015) and the Afghan Ministry of Public Health (399302). Throughout the study, any harm to the participants in the form of adverse events, serious adverse events, and participant deaths will be reported to ethics committees.

## Discussion

### Implementation

In total, six villages in five districts across two provinces were identified and agreed to participate in the study. A seventh village was approached but declined to be part of the study due to the randomisation process. A total of 1461 women were recruited and randomised into the study at baseline. Of the total recruited, 935 were currently married at baseline.

There are some important demographic differences between the districts sampled. In Kalakan district in Kabul, community members are predominantly Tajik with a small proportion of Pashtuns. In Nangarhar province (in both districts), community members are predominantly Pashtun with a small proportion of Tajiks. In Dashte Barchi district of Kabul province, community members are predominantly Hazara.

### Challenges

The research team faced unanticipated challenges in recruitment and randomization. Women were incredibly keen to be involved in the intervention, which posed challenges that the field team resolved through the development of additional processes to screen and manage participants.

The high demand to participate in the study and intervention led to very large numbers of women assembling for recruitment. This meant that adequately briefing them on the programme and study procedures in one large group was difficult and further complicated by women who continued to arrive throughout the day. In addition, crowd control was difficult with large numbers of women assembling for recruitment (up to several hundred at any time). As the day progressed, women became unsettled and in many cases aggressive due to fears of not entering the recruitment site before the recruitment quota had been reached. This often led to women finding ways to enter the recruitment location, for instance through windows or by pushing through doors, at which point recruitment became difficult and in some cases had to be terminated and continued on a different day.

Screening women for eligibility was also challenging. The majority of women did not have any form of identification proving their age (which is common in Afghanistan); therefore, much older women and younger girls attempted to be recruited despite the age criteria. Although attempts were made to make a judgment on age, this was often difficult. Furthermore, some women deemed ineligible to participate would return later in the day, or on the following day, often dressed in burqas or different clothing, or with someone else’s identity documents, in an attempt to pass the screening process. In addition, male family members occasionally came to the registration sessions, angry that the women in their families could not, or had not, been registered.

There were also a number of challenges in the randomization procedures. Much like for recruitment, managing large crowds was highly challenging and escalated by women sometimes being upset or angry by not being randomised into the intervention. At times, the randomization process had to be terminated and continued on another day due to concerns about the safety of WfWI and research staff. After several randomizations, it became evident that women had explained to other women what each colour signified, and some women would attempt to look into the box before drawing a ball to enable their selection of a blue ball. In these cases, women were asked to draw again.

To overcome these challenges, a number of procedures were implemented and these were modified throughout the recruitment to minimize risk and ensure study integrity. First, women were briefed in smaller groups of 20 (rather than one large group). Second, registration and randomization was conducted on the same day and quickly to minimize the number of women at the site. Third, only small numbers of women were let into the sites at a time with adequate staff at each point to monitor women’s entry and departure from sites. Finally, a permanent marker was used to mark women’s wrists to ensure they could not be registered twice in the study. These procedures eased the process and ensured the project could adhere to the protocol. At times, however, when the process became too challenging, the team stopped work for the day and returned the following day to maintain quality of process.

In one community, the research manager received a phone call from a husband of one of the study participants. The husband was asking questions about why the intervention was asking women about family violence. It became apparent that men in this community had found out about the questionnaire and the topics, including IPV, and were talking about it amongst themselves. The team paused fieldwork in this community and WfWI called a community meeting. The District Governor attended, with some men from the communities, and it was reiterated what the intervention was and hoped to achieve, reiterated that there were some questions about family conflict as these were about women’s health, but these were confidential and were part of a much broader set of questions. Following the meeting, the community agreed to allow the study to continue with no further similar issues.

### Baseline comparison

Despite the challenges of implementing research in a complex environment, where there was high demand for inclusion in the study and intervention, the baseline socio-demographic data shows comparability between study arms, showing randomization was effective (Table 2). There were no significant differences between study arms, either amongst all women (*n* = 1461), or amongst married women only (*n* = 935).

**Table 2:** Socio-demographic information by study arm

	All women						Currently married women only					
	Total	Intervention		Control		*p*-value	Total	Intervention		Control		*p*-value
		mean	(95% CI)	mean	(95% CI)			mean	(95% CI)	mean	(95% CI)	
*Age*	1461	29.3	28.7–29.9	29.3	28.6–30.0	0.99	935	32.4	31.8–33.0	32.4	31.7–33.1	0.97
		n	(%)	n	(%)			n	(%)	n	(%)	
*Relationship Status*												
Currently married	935	481	64.4	454	63.6							
Previously married	98	48	6.4	50	7.0							
Never married	428	218	29.4	210	29.4	0.89						
*Education*												
None	1129	581	77.8	548	76.8		781	408	84.8	373	82.2	
Madrasa	92	52	7.0	40	5.6		47	25	5.2	22	4.9	
Primary schooling	147	74	10.1	73	10.2		75	36	7.5	39	8.6	
Secondary schooling	93	40	6.4	53	7.4	0.31	32	12	2.5	20	4.4	0.38
*Borrowing food or money in past month because of not having enough*												
No	437	215	28.9	222	31.1		281	141	29.4	140	30.9	
Yes	1021	530	71.1	491	68.9	0.34	651	338	70.6	313	69.1	0.63

The average age of the respondents was just under 30 (29.3 intervention, 29.3 control). In both arms, just under two-thirds were currently married, and just under 30 % had never been married. Education levels in the whole sample were low: three-quarters of women had never received any formal education (77.8% intervention, 76.8% control). Of the quarter who had received education, around 6% had been to a Madrasa, 10% had received some form of primary school education, and 6.4% (intervention) and 7.4% (control) had some level of secondary education. There were also high levels of poverty, with approximately 70 % of the sample reporting borrowing food or money in the past month because they did not have enough.

There was similar balance amongst women who were currently married by study arm. Currently married women were slightly older than the entire sample (both married and unmarried) at about 32 years and this was balanced across arms. The overwhelming majority reported not receiving any schooling (84.8% intervention, 82.2% control). Only about 5% in either arm of married women had been to a Madrasa only, while about 8% had received some primary education and around 3% some secondary education. There were no significant differences between arms for married women in terms of borrowing money because of hunger, with around 70 % reporting this in each arm.

### Limitations

The study has a number of limitations, primarily linked to the wider challenges of operating in a conflict/post-conflict setting. Due to the challenges with recruitment and randomization at the start of the process, there are slightly more participants in the intervention arm (*n* = 747) compared to the control arm (*n* = 714). Despite this, the randomization was effective in achieving balance in socio-demographic measures across arms. The decision to randomize at the individual level, rather than the cluster level may have an impact. The concern remains that there could be some information diffusion from those in the intervention to those in the control group. WfWI had previously worked in two of the villages so there is some possibility that there could already be spillover effects in these villages, which may impact results.

We were advised not to include questions which might be considered offensive or very embarrassing for women. Therefore, we did not ask about sexual violence even though sexual violence in prevalent in Afghanistan [15] and measures of IPV include sexual violence in other settings. The low level of past-year sexual IPV reported in the Afghan DHS somewhat supports our decision, as it suggests that the notion was either hard to interpret culturally or particularly embarrassing for women to report.

Finally, while the study is currently operating, the ongoing security challenges of working in Afghanistan remain paramount. Depending on the wider political situation, this may reduce programming intensity and also lead to challenges around follow-up of participants.

## Conclusion

IPV remains a pressing concern in conflict, post-conflict and humanitarian emergencies, often eclipsing forms of state and non-partner sexual violence [1, 2]. The lack of well-evaluated interventions to prevent IPV is a major challenge in developing a strong evidence base on preventing IPV in these contexts. Despite the challenges of operating and undertaking research in complex settings, the implementation of this study shows that there is a significant desire for interventions and women’s education and empowerment in these contexts. The WfWI intervention trial in Afghanistan will provide important evidence on the scope and scale of IPV, as well as on the potential of economic and gender empowerment interventions to work effectively in complex settings.

## Footnotes

1 Bride price (*walwar* in Pashto and *sherbaha* in Dari) should not be confused with the dowry (*mahr*), the former which is a sum of money paid to the father or other male family member of a bride, and the latter a sum of money paid directly to the bride to provide economic security in case her husband dies or divorces here. Although *mahr* is enshrined in Islamic Sharia law, it is rarely implemented in Afghanistan with bride price being the most common practice.

2 This has been well documented by human rights organisations, for example: UN SCR 1325 Implementation in Afghanistan (AWN, 2011) http://www.awn-af.net/index.php/cms/content/501; AWN Annual 2nd Report on CEDAW in Afghanistan (AWN, June 2016); http://www.awn-af.net/index.php/cms/content/501; https://www.hrw.org/news/2016/05/25/afghanistan-end-moral-crimes-charges-virginity-tests; Their lives on the line: women human rights defenders under attack in Afghanistan (Amnesty International, April 2015) https://www.amnesty.org/en/documents/asa11/1279/2015/en/

3 See: www.whatworks.co.za
